# The Symptoms and Signs of Gallstone Disease1Read before the Esculapian Club, December 21, 1905.

**Published:** 1906-05

**Authors:** Francis W. McGuire

**Affiliations:** Buffalo, N. Y.; Attending Surgeon to Niagara Hospital


### The Symptoms and Signs of Gallstone Disease.[1]

BY FRANCIS W. McGUIRE, M. D., Buffalo, N. Y.
Attending Surgeon to Niagara Hospital.

THE symptomatology of gallstones is of exceptional interest because of its many and varied expressions. On the one hand stones may exist with few if any symptoms referable to the gall-bladder; while on the other they may produce such pronounced expressions as to permit of an immediate diagnosis. It is probably questionable that many cases can exist without producing any symptoms, because the closer study of these cases made possible by modern surgery, shows that the majority of these patients are sufferers from gastric and intestinal disturbances formerly classed as dyspepsia. Stones are supposed to be present in about 10 per cent of bodies examined on the post-mortem table. Reidel reports 10 per cent, Kehr 10 per cent, Brewer 12 per cent, while another writer states in elderly women, perhaps about every fourth person has gallstones. This estimate seems none too high, as stones may have been in a gall-bladder and

1. Read before the Esculapian Club, December 21, 1905.

passed out, and besides may be overlooked at an autopsy, unless it is conducted with this special purpose. In the majority of post mortem cases, the stones have never given rise to symptoms of sufficient severity to have caused them to be recognised during life. Gallstones that would have caused pain in passing through ducts have been found in feces, when no symptoms of their presence have been elicited within a recent period. The probable explanation of this is that there had been previous attacks and a fistula had formed between the biliary passages and the bowel. Moynihan reports such a case.

The infrequency of the recognition of gallstones requires emphasis, for it shows clearly enough that if gallstones can be brought to lie quietly in the gall-bladder, there may be a complete immunity from suffering; but this statement requires qualification, as it is an undoubted fact that the commoner manifestation of the presence of gallstones, which is indigestion, is never referred by the patient and rarely by the medical man to the gall-bladder or ducts. The most hasty examination into the history of operated cases, will show that in the large majority the earliest symptoms, and one that has caused at times a great deal of suffering, is indigestion. There is a variety of names given to this condition, all of which refer to trouble with the stomach, and not to the liver. It requires the unmistakable evidence of jaundice to associate the suffering with gallstones in the minds of the patients and in not a few medical men, yet jaundice is an inconstant and infrequent symptom of gallstone disease.

It is not accurate to say that gallstones in the majority of cases causes no symptoms. It must not be assumed that because there are no striking symptoms of the condition that there are no gallstones. In cases that come to operation, where undoubted symptoms are present only a short time, there will often be found evidence of chronic inflammatory processes that has taken years to accomplish. Gallstones cause symptoms in a great many cases where the true nature of the disease is never recognised. So, gallstones found at autopsy or upon a patient that has suffered for years with gastric disorder, may explain all the symptoms. The want of recognition of gallstone disease in its earliest stages must be insisted upon, for it is in this stage that surgical treatment should be advised if possible.

The symptoms and signs of gallstone disease that require discussion, are pain and colic, nausea and vomiting, jaundice, fever and tumor. Pain is either localised or referred; localised pain may be described as of two types: first, a dull aching pain due to increased tension or slight infection. Second, an acute intolerable pain which results from more severe infection. Pain is usually diffused over a large area, but tenderness is not especially marked. If, however, sudden pressure is made with the examining hand, there is a tightening of the muscles to protect the underlying structures from injury.

One of the best methods of eliciting tenderness is to hook the fingers up deeply under the costal arch, and ask the patient tô take a deep breath; when the sensitive gall-bladder reaches the examining fingers, the inspiration suddenly ceases, as if it were shut off. However, tenderness is often manifested by a tension of the right rectus. Pain that is diffused and aching in character is the variety that is confounded with stomach trouble. It is worse after eating and is, without exception, relieved by vomiting. This pain is due to increased tension caused by the impaction of the stone in the cystic duct, and as soon as stone falls back into gall-bladder, as it often does after vomiting, the pain is relieved.

When the pain is acute it is an expression of a higher degree of irritation and infection, involving the gall-bladder and ducts. This infection often extends to the peritoneum, causing a localised peritonitis. If the irritation is slight, the impaction is of brief duration and the infection set up is trivial and evanescent. When the impaction is prolonged, a cholecystitis or a cholongitis may be set up. The pain is more acute and the peritoneum investing the gall-bladder becomes more widely implicated, and adhesions more or less complex will be found. Referred pain is almost always associated with one or other of the foregoing. Pain may radiate to the right or left subscapular regions, rarely to the left; to the neck, the arm and to the epigastric region. Murphy says it is referred seven out of ten times to the right subscapular region, in obstruction of pelvis or gall-bladder or cystic duct; in one case to the left subscapular region, and in the other to the chest as high as the neck.

There is an area of tenderness described by Boas on the right side behind, on a level with the twelfth dorsal vertebra, two or three fingers breadth from the spine. Moynihan considers this a sign of great value, and asearch for this tenderness is a necessary part of the examination of all patients who suffer or are suspected to suffer with gallstone disease.

*Colic.* The pain experienced as the result of gallstone irritation is often colicky in character; since in the vast majority of patients, a stone never enters on its travels from the gall-bladder, it is quite clear that hepatic colic is a far less frequently observed symptom than the dull or more acute localised pains. Colic is therefore caused by an overexertion of the muscular wall of the gall-bladder or ducts, in the onward passage of a foreign body.

It occurs when the foreign body is in transit, as soon as the body passes out of the duct; or if it becomes fixed, the muscular effort slackens, and finally ceases, and the ducts proceed to adapt themselves to the intruder. A small stone may pass along the ducts without causing pain.

Murphy has called attention to the fact that a stone in its backward movement can cause colic. For example, after a cholecystostomy, when a stone impacted in the cystic duct works its way backward into the gall-bladder it is evident, therefrom, that gallstones cause pain,—first, by causing irritation and infection. Secondly, by traversing the ducts with a foreign body, setting up a spasm of the muscular wall of gall-bladder or ducts. There is considerable difference of opinion regarding the cause of colic; some maintain it is due to infection, others that it is due to spasms of the gall-bladder or ducts and is an indication that a foreign body is in transit through the ducts. My own views correspond with the latter.

*Nausea and Vomiting.* These are a common manifestation of gallstones, and it is these symptoms that causes the stomach to be unjustly labelled the organ at fault. There is undoubtedly indigestion present, and has as its natural sequence an attack of nausea and vomiting which brings relief. Nausea and vomiting are partly reflex in origin and partly due to direct irritation of the stomach.

The feeling of deadly sickness and the vomiting which follows are due to momentary or enduring impaction of stone in the cystic duct. It is the obstruction which reflexly produces the nausea and vomiting. The vomiting when prolonged causes general muscular relaxation, and in this condition the stone is allowed to fall back into the gall-bladder. In all patients suffering from constant attacks of nausea and vomiting, it is desirable that the existence of cholelithiasis should be borne in mind. The examinations should include an attempt to elicit posterior tenderness mentioned above.

*Jaundice.* Jaundice is unhappily an infrequent and often an inconspicuous symptom of cholelithiasis. If it occurred more frequently, there would be earlier and more frequent recognition of the disease. If gallstones have a normal habitat at all, it is in the gall-bladder, and consequently entirely apart from the bile current; but if present, jaundice occurs as a complication in several different ways. First: stones in the gall-bladder may set up a cholecystitis, and the infection travels to the common duct, producing a cholangitis with jaundice. Second, a peritonitis may be established, causing pressure on the common duct. Third, a stone may lodge in the cystic duct, causing pressure on the common duct. Fourth, impaction of stone in hepatic or common duct. Fifth, infection may extend to the pancreatic ducts, causing chronic, pancreatitis with jaundice. From this we may readily see how infrequent jaundice attends this disease. It is reported to occur in about 12 per cent to 15 per cent of cases.

The presence of pain preceding jaundice is considered by some important as it is then caused by cholelithiasis. In this condition the jaundice appears gradually and deepens according to the completeness of the obstruction. After the obstruction is relieved the jaundice passes gradually away. If, however, a stone is impacted and the ducts become dilated behind it, a ball valve action results. In this condition the jaundice will be remittent or at least will vary in the depth of its color. When jaundice appears painlessly, it is almost always due to malignant disease. It increases gradually day by day without remission until the skin is a greenish-yellow. Moynihan states there is a decided difference in the color of jaundice due to malignant and non-malignant causes. In the former, green predominates; in the latter, golden yellow. The importance of associated distention of the gall-bladder with jaundice was pointed out by Courvoisier and later confirmed by Mayo Robson, Cabot and others. Courvoisier says: "In cases of chronic jaundice due to blockage of the common duct a contraction of the gall-bladder signifies that the obstruction is due to a stone; in a dilatation of the gall-bladder the obstruction is due to other causes than stone." This statement, however, does not hold true in all cases, but seems to do so in about 90 per cent.

*Fever.* The temperature in this condition is interesting and depends upon the anatomical arrangement of the lymphatic system. The gall-bladder has no lymphatic nodes, and few lymphatic channels. There is one gland at the junction of the gall-bladder and cystic duct; another at the entrance of the cystic duct into the comon duct, and a small number closely associated with the common duct. Consequently, while the infection is confined to the gall-bladder, we have little or no temperature, but when the infection extends to the cystic duct, we may have a temperature of 102° or 103°. If the infection extends still further to the common duct, we may have a temperature reaching as high as 104° or 105°, showing the influence of free and lymphatic absorption.

The temperature in these conditions rises suddenly, lasting a few hours, recurring at irregular intervals of hours or days. A temperature chart showing abrupt peak-like elevation, with normal interspace is quite characteristic of infection of the billiary ducts. In later stages when the infection has extended to the finer bile channels, the temperature may show no remissions, the temperature ranging from 103° to 105°, never returning to normal.

*Tumor.* A tumor of the gall-bladder in gallstone disease is caused by a stone or stricture in the cystic duct. When a stone or stricture is present in the common duct—in rare cases when the gall-bladder is crowded with stones—it may also occur by the enlargement of a lymphatic node, or by torsion or flexion at the neck of the gall-bladder. It is produced also when there is obstruction to the common duct by enlargement, simple or malignant, of the head of the pancreas. When a stone is impacted in the pelvis of the gall-bladder or cystic duct, the gall-bladder distends behind the obstruction; the fluid at first may be deeply tinged with bile, but soon all traces of coloring matter disappears, and a condition of hydrops exist. If this fluid becomes infected, the condition of empyema of the gall-bladder is recognised.

A distended gall-bladder containing bile is due to pressure upon the common duct by growths or chronic inflammation of the pancreas. A tumor of the gall-bladder may be due to malignant disease of the gall-bladder itself which, in the majority, of instances, is the late result of gallstone irritation.

The tumor formed by enlarged gall-bladder is generally easily palpated, and at times visible to inspection. As a rule the swelling is tender and a feeling of nausea is produced by handling it. The edge of the liver is felt above and the colon on inflation is recognized below. A large mass of adherent omentum over the gall-bladder may blur the outline of the tumor. It may also be blured by small intestine or a colon being adhered to liver or gall-bladder. A tumor of the gall-bladder will move upward during inspiration, and cannot be held down. Other tumors, those for example of the kidney, stomach, colon and omentom, can be held unless adherent to the liver or diaphragm.

There is no doubt but that the diagnosis of gallstones from other conditions producing pain, localized or general, within the abdomen, radiating to chest or back with vomiting and collapse is attended with a great deal of difficulty and at times is impossible. Nevertheless, the diagnosis is often made readily and with certainty. In those obscure cases we may not be able to make a pathological diagnosis, but we can a surgical one. We can demonstrate beyond question that the cause of the symptoms is one of several conditions, all of which are surgical, and this being the case, operation should be resorted to before the gross pathological changes make the diagnosis more certain and the prognosis less favorable. I do not wish it to be inferred that we should make reckless resort to the knife, for exploratory purposes, but should avail ourselves of all possible diagnostic measures, for it is also a false conservatism which stands in the way of early operative interference in gallstone disease.

1175 MAIN STREET.

